# Risk factors associated with mental illness in Oyo State, Nigeria: A Community based study

**DOI:** 10.1186/1744-859X-4-19

**Published:** 2005-12-22

**Authors:** OE Amoran, TO Lawoyin, OO Oni

**Affiliations:** 1 Department of Community Medicine, University College Hospital, Ibadan, Nigeria; 2 Office of Medical officer of Health, Saki East Local Govt Area, Oyo State, Nigeria

## Abstract

**Background:**

The main objective of this study was to determine the prevalence and factors associated with mental illness in Oyo State at community level using the general health questionnaire as a screening tool.

**Method:**

This cross-sectional, community- based survey was carried out among adults in three randomly selected LGAs using multi-stage sampling technique.

**Results:**

A total of 1105 respondents were assessed in all. The overall prevalence of psychiatric morbidity in Oyo state Nigeria was found to be 21.9%, (18.4% in the urban areas and 28.4% in the rural areas, p = 0.005). Young age ≤ 19 yrs (X^2 ^= 20.41, p = 0.00013), Unemployment (X^2 ^= 11.86 p = 0.0005), living condition below average (X^2 ^= 12.21, p = 0.00047), physical health (X^2 ^= 6.07, p = 0.014), and large family size (X^2 ^= 14.09 p = 0.00017) were associated with increase risk for psychiatric morbidity.

Following logistic regression analysis, Unemployment (C.I = 1.18–3.70, OR -2.1) and living conditions perceived to be above average (C.I = 1.99–5.50, OR-3.3) were significant predictors of mental illness while family size less than 6 (C.I = 0.86–0.97, OR-0.91) was protective.

**Conclusion:**

The teenagers and the rural populations are in greater need of mental health promotional services. Family planning should be made freely available in order to reduce the family size and hence incidence of mental illness in the African population.

## Introduction

Mental health is defined as the capacity to work, capacity to love and the capacity to play and for recreation [1]. Approximately one in five of the world's youth, 15 years and younger suffer from mild to severe mental disorders. A large number of these children remain undetected and untreated [2]. It must be noted that mental health is one of the more recently added components of Primary Health Care (PHC) and means more than merely the presence or absence of obvious mental illness. In Nigeria 28.5% of those attending primary care setting in an urban area were found to have psychiatric morbidity [3,4]. The disintegration of the traditional, extended family due to factors such as economic migration inevitably creates socio-cultural changes that may affect the mental health of the individuals in the society. Furthermore, concerns for job security and the economic survival of the household can create enormous pressure on individuals which may in turn affect their mental health [5,6].

Available studies have been largely facility-based, while community- based studies have been very scanty. Several studies have been done using general health questionnaire (GHQ12) as a tool for screening mental illness in developed countries where the prevalence of psychiatric morbidity ranges between 17%–25% [6-8]. This study assessed the prevalence of and factors associated with mental illness in Oyo State at community level using the general health questionnaire as a screening tool. The public health significance of this study is that it will provide a conceptual framework for addressing mental health promotion goals. It also offers a highly appropriate framework through which community-based mental health activities could be addressed. Moreover, it could also be used for health planning and practice.

## Materials and methods

The study was carried out in Oyo State one of the 36 states in Nigeria. This community based study is cross sectional in design and aimed at collecting data on mental health in rural and urban Oyo State, Nigeria.

### Sampling procedure

Multistage sampling technique was used to obtain a representative sample of the communities in Oyo state. The communities where the study was carried out were chosen as follows:

#### Stage 1

A sampling frame of all the local government areas in Oyo state was drawn and stratified into urban and rural areas based on World bank classification [9]. One rural and two urban local government areas was obtained by simple random sampling (balloting). This is based on the fact that two thirds of Oyo state is urbanized. Ibadan North-West, Egbeda and Saki-East local government areas were selected.

#### Stage 2

Sampling frame of all the communities in the selected local government areas was drawn. The communities where the study was carried out were randomly selected by simple random sample (balloting). The communities selected were Idikan in Ibadan North-west LGA, Olubadan Estate in Egbeda LGA and Ago-amodu in Saki-East LGA.

#### Stage 3

Using the PHC house numbering where available (in places where it has not been done the houses were numbered for the purpose of the study). Systematic sampling technique was employed to select the houses that were visited in the chosen communities. Seventy-four houses were selected in Idikan, eighty-five houses in Olubadan Estate and ninety-eight houses in Ago-amodu.

#### Stage 4

One household in each of the houses selected were recruited into the study.

#### Stage 5

Every resident aged 15 years and above who has resided in the area for at least 6 months was interviewed in the households selected. A total of one thousand, one hundred and five subjects were recruited into the study.

A sample size formulae for comparing two proportions was used to obtain the sample size. Prevalence of 12.0% of poor mental health using GHQ 12 questionnaire among clinical students of University of Ibadan was used as estimate for urban community, while the prevalence of 21.3% among rural primary health care patients in Nigeria was used for rural community [4,5]. A precision of 95% is desired with a power of 90%. The calculated sample size was 610 while this was doubled to 1220 with response rate of 90.6% (1105 responses).

### Data collection

The study was conducted using an interviewer administered structured questionnaire. The GHQ-12 was used to assess mental health status of the respondents. Scores were calculated with a 0-1-1 scale with a maximum score of 1 and a minimum score of 0 for each item. A score of three or more was used as cut-off to classify into good and poor mental health. WHO quality of life questionnaire is a five point scale with items which ranged in rating from "very poor", "not at all" or "very dissatisfied" (1 point) to "very good", "extreme amount" or "very satisfied" (5 points). For items with reverse scores "not at all" was scored 5 and "extreme amount" was scored 1. The score for both mentally healthy and mentally ill was computed to asses the effect of psychiatric morbidity on quality of life.

This questionnaire was translated into the local language for easy administration and translated back to English to ensure accuracy of translation. The GHQ (12) and WHO quality of life questionnaires were administered by research assistants after adequate training and the author's supervision. Research assistants were recruited from the communities and were trained to administer the questionnaires. The research assistants were recruited based on minimum qualification of OND certificate. They were trained on how to extract the information on psychiatric symptoms and their relevance to the research work. They were also guided through a good number of questionnaires until a reasonable level of competence had been attained before being left to do it themselves.

Data generated in the study were manually cleaned and then entered into the Computer using SPSS 10 statistical software for analysis. Logistic regression analysis was done to determine factors associated with mental ill health in rural and urban Oyo State and also to remove the effect of confounding variables. The dependent variable was psychiatric morbidity as a dichotomous variable with a score of less than 3 being an option indicating good mental health while a score of 3 and more being another option indicating abnormal or psychiatric morbidity. All the variables which were significant in the bivariable analysis with a p < 0.08 were fed into the model. Odd ratios were adjusted and p values of <0.05 were taken as significant for the study.

## Results

### Demographic characteristics

Psychiatric morbidity was more prevalent in the rural population (28.4%) compared with the urban population (18.4%) (X^2 ^= 3.69 p = 0.005). The adolescents in this study (15–19 yrs) had the highest prevalence of psychiatric morbidity (43.7%, p = 0.00013) in Oyo state. The indigenes, that is, the Yorubas were more mentally stable when compared with the migrant tribes (the Ibos, Hausas and other minority tribes) (21.2% vs 33.3% p = 0.253). Females had higher prevalence of psychiatric morbidity (24.2% vs 18.1%, X^2 ^= 0.83 p = 0.36).

### Family characteristics

The Single (never married & separated and divorced) had a higher morbidity rate when compared with the married (p= 0.091). This is shown in table 2. Fewer respondents living within the extended family structure had higher prevalence of mental ill-health when compared with those living within nuclear family structure, (X^2 ^= 0.09, 1df p = 0.766). Those living within monogamous family structure had higher prevalence for psychiatry morbidity. (X^2 ^= 0.23, p = 0.634). Small sized families were significantly mentally healthier than large size families (X^2 ^= 14.09 p = 0.00017).

**Table 1:** Socio-demographic characteristics and Mental Health Status

**Characteristics**	**Total No & (%)**	**Psychiatric Morbidity No & (%)**
**Age**		
15–19	197 (17.8)	86 (43.7)
20–39	529 (47.3)	92 (17.4)
40–64	304 (28.0)	51 (16.8)
>65	75 (6.9)	13 (17.3)
	**1105 (100.0)**	**242 (21.9)**

**Sex**		
Male	415 (37.6)	75 (18.1)
Female	690 (62.4) **1105 (100.0)**	167 (24.2) **242 (21.9)**

**Tribe**		
Indigenes	1045 (94.6)	222 (21.2)
Non- Indigenes	60 (5.4)	20 (33.3)
	**1105 (100.0)**	**242 (21.9)**

**Location**		
Urban	721 (65.3)	133 (18.4)
Rural	384 (34.7)	109 (28.4)
	**1105 (100.0)**	**242 (21.9)**

**Table 2:** Family characteristics and Mental Health Status

**Characteristics**	**Total No & (%)**	**Psychiatric Morbidity No & (%)**
**Marital Status**		
Single (Never Married)	447 (40.4)	125 (28.0)
Married	564 (51.0)	89 (15.7)
Separated, Divorced or Widowed	94 (8.6)	28 (29.8)
	**1105 (100.0)**	**242 (21.9)**

**Type of family**		
Nuclear	554 (50.1)	108 (19.5)
Extended	551 (49.9)	114 (20.7)
	**1105 (100.0)**	**242 (21.9)**

**Type of Marriage**		
Monogamy	542 (49.0)	112 (20.7)
Polygamy	563 (51.0	130 (23.1)
	**1105 (100.0)**	**242 (21.9)**

**Family size**		
<6	793 (71.8)	126 (15.9)
>6	312 (28.2)	116 (37.2)
	**1105 (100.0)**	**242 (21.9)**

### Socio- economic characteristics

The unemployed had the highest prevalence for psychiatric morbidity when compared with the employed (X^2 ^= 11.86 p = 0.00058). Among those employed, the senior professionals had the highest unadjusted psychiatric morbidity rate (23.2%) while the students had a relatively high prevalence of mental ill-health (37.3%) when compared with others that are employed. Educational level was collapsed into two groups (high and low). Respondents with high level of formal education (secondary level and above) had a higher morbidity rate than those with low level of formal education (Nil and primary) (X^2 ^= 0.97, p = 0.325).

The respondents who perceived their living condition to be above the average for their status were more mentally stable compared with those who did not (X^2 ^= 8.13 p = 0.0043). Those with chronic mental illness had a higher prevalence of psychiatric morbidity (X^2 ^= 6.07 p = 0.014). Furthermore, those with good social relationship were more mentally stable (X^2 ^= 8.13 p = 0.0043).

### Multivariate logistic analysis

Table 4 shows the adjusted odds ratio and the confidence interval for the risk of mental illness in Oyo State. Family size greater than 6 (p = 0.002), reported living conditions above average (p = 0.0001) and unemployment (p = 0.011) increased the risk of mental ill health. The presence of physical illness (p = 0.056) was of borderline significance for mental illness.

**Table 3:** Socio-economic characteristics and Mental Health Status

**Characteristics**	**Total No & (%)**	**Psychiatric Morbidity No & (%)**
**Occupation**		
Senior Professional	95 (8.6)	22 (23.2)
Junior Professional	103 (9.3)	22 (21.4)
Skilled	353 (31.9)	64 (18.1)
Unskilled	400 (36.2)	67 (16.8)
Students	75 (6.2)	28 (37.3)
Unemployed	79 (7.1)	39 (49.4)
	**1105 (100.0)**	**242 (21.9)**
**Job Status**		
Employed	924 (92.9)	203 (21.9)
Unemployed	79 (7.1)	39 (49.4)
	**1105 (100.0)**	**242 (21.9)**

**Education**		
Low	475 (43.0)	90 (18.9)
High	530 (57.0	152 (24.1)
	**1105 (100.0)**	**242 (21.9)**

**Physical health**		
Poor	538 (48.7)	155 (28.8)
Good	567 (51.3)	87 (15.3)
	**1105 (100.0)**	**242 (21.9)**

**Social health**		
Poor	599 (54.2	183 (30.5)
Good	506 (45.8)	59 (11.7)
	**1105 (100.0)**	**242 (21.9)**

**Living condition**		
Below average	570 (51.6)	195 (34.2)
Above average	535 (48.4)	47 (8.8)
	**1105 (100.0)**	**242 (21.9)**

**Table 4:** Adjusted Odds Ratio for the Risk of Mental Illness in Oyo State

**Variables**	**Odd Ratio**	**Confidence Interval**	**P-value**
**Marital status**	1.34	0.84–2.10	0.22
Not Married	1.00		
Married			
			
**Family size**	0.91	0.86–0.97	<0.0005
<6	1.00		
>6			
			
**Age**	0.72	0.18–2.90	0.66
15–19	1.58	0.80–3.10	0.19
20–39	1.24	0.78–1.90	0.36
40–64	1.00		
>64			
			
**Location**	0.53	0.22–1.28	0.158
Urban	1.00		
Rural			
			
**Living Condition**			
Below average	3.30	1.99–5.50	0.001
Above average	1.00		
			
**Socio-economic class**			
unemployed	2.10	1.18–3.70	0.01
Low	1.40	0.91–2.15	0.12
High	1.00		
			
**Social Health**	1.32	0.77–2.27	0.31
Below average	1.00		
Above average			
			
**Physical Health**	1.58	0.99–2.52	0.06
Below average	1.00		
Above average			

## Discussion

This study examined the prevalence of psychiatric morbidity in the urban and rural areas of Oyo state with a view to identify the factors that are associated with mental illness in the general population at the community level. The overall prevalence of psychiatric morbidity found at the community level in this study was 21.9%. It is however slightly higher than what is found in other community-based surveys carried out in developed countries such as Spain, Canada, Norway and Australia [9-11].

The prevalence of psychiatric morbidity in the rural area was found to be significantly higher than the prevalence in the urban location. This is contrary to what was found in similar studies carried out in developed countries such as Great Britain where mental disorder was commoner in the urban areas than in the rural population [12]. The adolescent age group was found to have higher psychiatric morbidity when compared to the adults. Similar observations have been made in several studies [21,22]. The adolescent period is a turbulent period in life when there is transition into adulthood and self autonomy. This may explain the higher morbidity rates [23].

The study underlines the effect of family structure on the mental health of the population. Marriage was found to be associated with mental stability in Oyo state. Those separated from their spouses, divorcees and widows had a higher mental morbidity. Sticking to acceptable family structures may create mental tension in the communities studied. Aspiration to meet up to the community standards is usually a common source of mental stress. [14,15]. The indigenes were found to be more mentally stable than non-indigenes showing that migrants in Nigeria may be predisposed to setbacks psychologically when compared with the indigenes. This needs however to be further investigated as this is contrary to research done among Canadian Chinese migrants which shows that they do not suffer mentally compared with the general Chinese population [15].

Large family size and Unemployment was found to be associated with increase in psychiatric morbidity. This study corroborates the findings of several authors who found out that the larger the size of the family the lower the quality of child upbringing. This may lead to delinquent behavior among the children and increased mental stress on the care providers [16,17]. Unemployment is an important risk factor for mental illness and a significant determinant in the development of mental pathology especially among the adolescents [18]. However among those with employment the professionals had the highest morbidity rate. The possible reason for this in a Nigerian population is not immediately clear. Those with high level of formal education had higher psychiatric morbidity rate in the Nigerian community. This is similar to the conclusion of many authors [19,20].

This study shows that prevalence of psychiatric morbidity is high in Oyo state, Nigeria and slightly higher than what is obtained in the community- based studies in the developed countries. The rural population is in greater need of mental health promotional services. Basic essential needs provided by the government in both rural and urban areas especially made available to the younger generation and promotion of family planning to reduce family size would help to reduce psychiatric morbidity and improve quality of life in this African population.
